# Networks that Govern Cardiomyocyte Proliferation to Facilitate Repair of the Injured Mammalian Heart

**DOI:** 10.14797/mdcvj.1300

**Published:** 2023-11-16

**Authors:** Daniel J. Garry, Jianyi (Jay) Zhang, Thijs A. Larson, Hesham A. Sadek, Mary G. Garry

**Affiliations:** 1 University of Minnesota, Minneapolis, Minnesota, US; 2 NorthStar Genomics, Eagan, Minnesota, US; 3 University of Alabama at Birmingham, Alabama, US; 4 UT Southwestern Medical Center at Dallas, Dallas, Texas, US; 5 University of Minnesota, Minneapolis, MN

**Keywords:** cardiomyocyte proliferation, cell cycle regulators, Hippo signaling, SHH signaling, hypoxia, metabolic factors, myocardial injury

## Abstract

Cardiovascular diseases are the number one cause of death worldwide and in the United States (US). Cardiovascular diseases frequently progress to end-stage heart failure, and curative therapies are extremely limited. Intense interest has focused on deciphering the cascades and networks that govern cardiomyocyte proliferation and regeneration of the injured heart. For example, studies have shown that lower organisms such as the adult newt and adult zebrafish have the capacity to completely regenerate their injured heart with restoration of function. Similarly, the neonatal mouse and pig are also able to completely regenerate injured myocardium due to cardiomyocyte proliferation from preexisting cardiomyocytes. Using these animal models and transcriptome analyses, efforts have focused on the definition of factors and signaling pathways that can reactivate and induce cardiomyocyte proliferation in the adult mammalian injured heart. These studies and discoveries have the potential to define novel therapies to promote cardiomyocyte proliferation and repair of the injured, mammalian heart.

## Introduction

Cardiovascular disease is the number one cause of death worldwide and in the US.^1^ A number of known and unknown factors can perturb the heart, resulting in an injury that commonly progresses to heart failure with reduced ejection fraction (HFrEF). Several of these stressors include ischemia (coronary artery disease), inflammation (viral mediated myocarditis such as SARS-CoV-2),^2^ cardiomyocyte membrane instability (dystrophic cardiomyopathy such as patients with Duchenne muscular dystrophy),^3,4^ hypertension,^5^ diabetes mellitus, obesity,^6^ infectious agents (ie, Chagas myocarditis),^7^ genetic mutations,^8^ structural heart defects, toxins (alcohol or recreational drug use),^9^ and others. As a result of these stressors, one in three Americans have cardiovascular disease and more than 7 million Americans are living with heart failure.^10^ Despite the implementation of guideline-directed medical therapy and device therapy (cardiac resynchronization therapy, defibrillator therapy, ventricular assist devices, etc), approximately 50% of patients living with heart failure will not survive more than 5 years from their initial diagnosis.^11^ The only curative therapy for patients with end-stage heart failure is orthotopic heart transplantation.^12^ Unfortunately, this curative therapy is severely limited by the lack of sufficient numbers of donor organs. While it is estimated that more than 100,000 Americans would benefit from cardiac allotransplantation, only about 3,500 patients are recipients.^13^ Therefore, there is a need to explore alternative strategies to promote cardiac repair and regeneration. One strategy that has received intense interest has focused on deciphering the signals and the networks that promote cardiomyocyte proliferation.^14,15,16^ The ability to generate more cardiomyocytes following an acute injury or insult would decrease cardiac remodeling and the progression to heart failure. Here in this review, we will focus on the use of model organisms and the recent discoveries that hold tremendous promise for the promotion of cardiomyocyte proliferation and cardiac regeneration.

## Cardiac Regeneration in Lower Organisms

**Figure 1:**
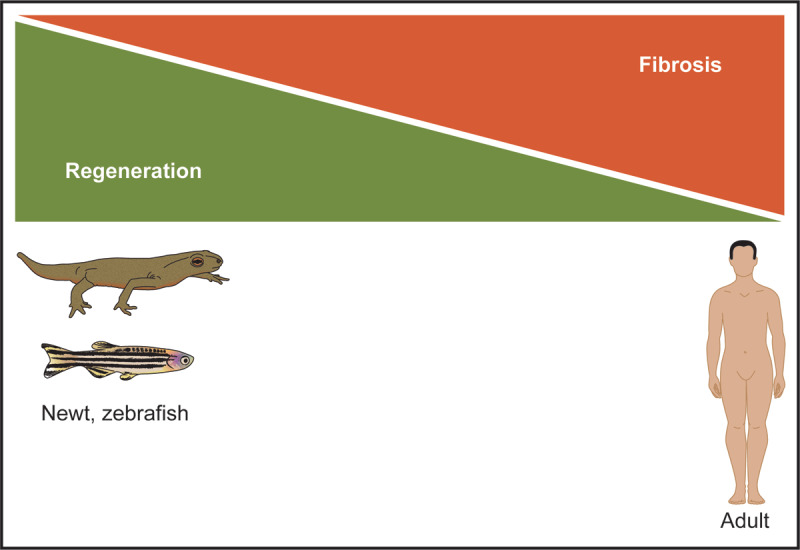
Lower organisms have a remarkable regenerative capacity following myocardial injury. The adult newt and zebrafish are examples of lower organisms that can completely regenerate their hearts following an acute injury. This regenerative process is due to cardiomyocyte dedifferentiation and proliferation in the absence of a fibroproliferative (ie, scar) response. In contrast, the adult mammalian heart has a very limited regenerative capacity, and repair of the adult heart is associated with extensive fibrosis in order to stabilize the injured heart. Figure is adapted from references 17-20.

Previous studies have characterized the remarkable reparative response of the invertebrate heart in response to an injury that results in cardiomyocyte proliferation, decreased fibrosis (scar) and improved cardiac function. This reparative process is evident in lower organisms such as the newt and zebrafish. For example, the newt or salamander (semi-aquatic amphibians) is able to regenerate a number of body parts including the amputated limb, the mandible, the retina, and the heart.^17^ This remarkable capacity is clearly evident as the amputated limb forms a blastema at the site of injury and progresses to “rebuild” its muscular and skeletal architecture with digits and function that is indistinguishable from its other limbs.^17^ This temporal and spatial memory that is required for the formation of a perfectly symmetrical and functional limb remains incompletely defined. Similarly, the newt adult heart has the capacity for regeneration. Amputation of the ventricular apex is followed by the formation of a thrombin clot. The blastema is then formed at the border of the injured heart, which promotes the dedifferentiation of cardiomyocytes and inflammatory response characterized by macrophages that begin to phagocytose the necrotic tissue. The dedifferentiated cardiomyocytes then begin to proliferate and, over a 6- to 8-week period, will completely restore the cellular and structural architecture of the injured heart, resulting in normal function and an absence of fibrosis (Figure 1).^18,19^ Studies have focused on the definition of pathways that may govern this regenerative response in the injured newt heart. Transcriptional and proteomic profiling of the injured newt heart revealed that inflammatory pathways and signaling pathways (ie, transforming growth factor beta signaling pathway, fibroblast growth factor pathway, bone morphogenetic protein pathway, sonic hedgehog (SHH) pathway, and others) were dynamically regulated during this reparative process.^17,20,21^ Moreover, Singh et al. demonstrated that inhibition of the SHH pathway using the small molecule inhibitor, cyclopamine, completely abolished the cardiomyocyte proliferative response and cardiac regeneration in the injured newt heart.^18^ Furthermore, long-term organ cultures of newt hearts in vitro underscored the importance of dedifferentiation of the cardiomyocytes and cell cycle reentry resulting in cellular proliferation.^22^ Additionally, the newt heart has a distinct anatomy (3-chambered heart and limited wall thickness to promote diffusion of nutrients from the circulating blood) and is composed of primarily mononucleated cardiomyocytes (> 95%) compared with 25% in mice and 75% in the human heart. While the adult newt heart has a tremendous regenerative capacity, the inability to use genetic technologies (gene knock-in, gene disruption, transgenesis, fate mapping strategies), the aquatic habitat, and the absence of a sequenced genome collectively limit the research using this unique regenerative animal model.

An alternative research model is the zebrafish model (Figure 1), which has largely replaced the newt. Like the newt, zebrafish have a remarkable regenerative capacity as they can completely repair the injured heart, fins, kidney, spinal cord, retina, and other organs. The zebrafish genome has been sequenced to facilitate the engineering of transgenic and gene disruption models. Moreover, zebrafish are relatively transparent, which facilitates phenotypic screens. The zebrafish heart is a 2-chambered heart (single ventricle and single atrium) and has tremendous regenerative capacity.^23^ Previous studies, using transgenic fate mapping strategies, demonstrated that amputation of the ventricular apex of the zebrafish heart resulted in cardiomyocyte proliferation and repopulation of the amputated chamber, with complete restoration of architecture and function within 60 days of the injury.^24^ Importantly, these injuries lacked a fibroproliferative response and, therefore, essentially no scar was associated with the regenerated heart. Moreover, genetic disruption studies established the essential role of networks including NF-κB, ErbB2, vitamin D, Notch, BMP10, Neuregulin, Wnt, and others.^24^ Additionally, the endocardium and epicardium provide important cues in addition to the vascularization and innervation that collectively promote cardiomyocyte proliferation. Like the newt, > 95% of the cardiomyocytes are mononucleated, and uniformly these cells may all be capable of reentering the cell cycle in response to appropriate cues. Transgenic fate mapping strategies support the notion that all the newly formed cardiomyocytes are derivatives of preexisting cardiomyocytes. The overall goal for studying the regenerative pathways associated with cardiac injury in lower organisms (such as the newt and zebrafish) is the notion that evolutionary conserved pathways may serve as a key to unlock the gateway that regulates tissue regeneration in the mammalian organism.^18^

## Neonatal Cardiac Regeneration in the Mammalian Heart

Studies undertaken by the Field’s laboratory using thymidine incorporation assays defined a second wave of cardiomyocyte proliferation that peaked on P4.6 days and continued to decrease thereafter.^25^ Twenty years later, the Sadek lab at University of Texas Southwestern Medical Center at Dallas demonstrated that cardiac injury (amputation of the apex of the left ventricle or left anterior descending (LAD) or LAD coronary artery ligation injury) in the P0 to P2 neonatal mouse heart could completely regenerate as a result of proliferating cardiomyocytes that were derivatives of preexisting cardiomyocytes.^26^ This regenerative window was limited because P7 cardiac injuries were unable to regenerate and were associated with a robust fibroproliferative response. The cardiomyocyte proliferation in the neonatal heart was shown to be enhanced by Meis1 deletion, hypoxia, and decreased oxidative stress.^27^ These and subsequent studies demonstrated that neonatal cardiac regeneration was dependent on the transition from mononucleated to binucleated cardiomyocytes, the metabolic transition of the neonatal heart, oxygen signaling, DNA damage response(s), and transient molecular mechanisms that govern key signaling pathways.^26,27^

Recent studies performed by the Zhang laboratory demonstrated that apical resection of the left ventricle of the P1 piglet was associated with cardiomyocyte proliferation and complete restoration of the cardiac architecture (without the presence of scar), but this response was even more limited than the mouse since later injuries were not associated with repair and regeneration.^28^ Follow-up studies, which included a second injury on P28, also demonstrated complete restoration of the ventricle and function.^29,30^ These latter results using sequential injuries (P1 and P28) are consistent with the notion that cycling cardiomyocytes were responsible for the repair and regeneration of the second injury. Collectively, these results support the evolutionary conservation of pathways and factors that promote cardiomyocyte proliferation and support the notion that these pathways or factors may be harnessed to promote repair of the injured adult mammalian heart.^15,16,18^

## Cardiomyocyte Turnover or Proliferation in the Adult Mammalian Heart

Adult mammalian tissues and organs have variable regenerative potential. At one extreme is adult mammalian skin, liver, bone marrow, skeletal muscle, and other tissues and organs that have an extremely high regenerative capacity following injury. For example, an injury that destroys up to 90% of skeletal muscle is associated with a highly regenerative response resulting in complete restoration of the cellular architecture and preserved function within a one- to two-month period.^31,32,33,34,35^ On the other extreme are organs such as the heart and brain that have a very limited regenerative potential.

**Figure 2:**
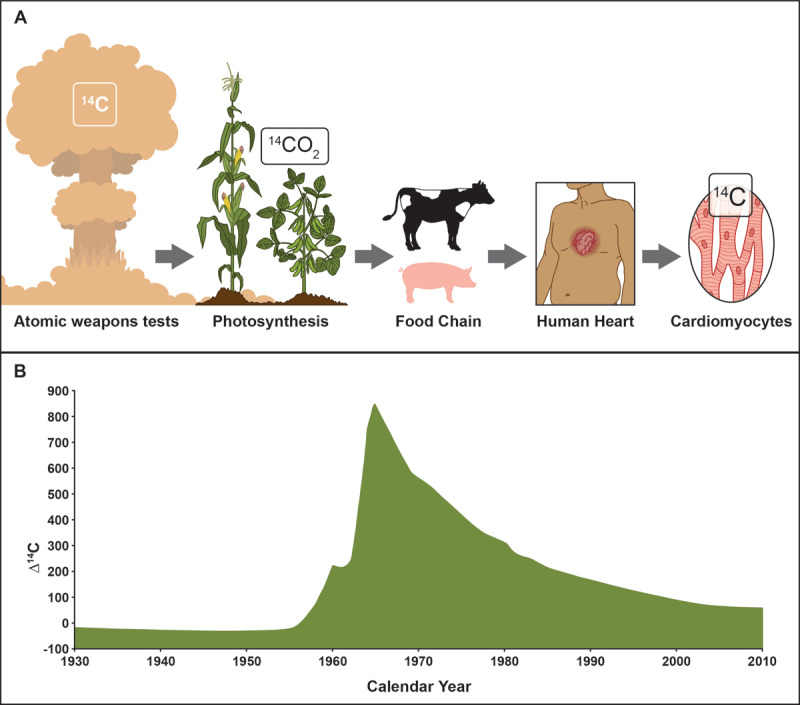
The adult human heart has a low turnover of cardiomyocytes. **(A)** Radiocarbon dating of the human heart relies upon a pulse **(B)** of ^14^C via the aboveground atomic tests, which was then incorporated into vegetation (via photosynthesis) and consumed by animals and humans. The levels of ^14^C found in cardiomyocyte nuclei were used to determine the birth date of the cardiomyocytes. These studies support the notion that 0.5% to 1.0% of human cardiomyocytes turn over each year. While this number is relatively low, these studies emphasize that pathways and subpopulations of cells are present and could be enhanced as a therapeutic initiative. Figure is adapted from references 36–38.

Previous studies have examined cardiomyocyte turnover using an array of techniques including the quantification of mitotic figures in the adult heart, incorporation (pulsing) of DNA nucleosides such as 5-bromo-2’-deoxyuridine or quantification of mononucleated or binucleated cardiomyocytes. Subsequently, Friesen’s laboratory undertook an analysis of human cardiomyocyte turnover using radiocarbon dating,^36^ a technique that was readily established and used extensively to date fossils. The technique was based on the concept of ^14^C generation in the atmosphere that was incorporated into plants/vegetation (via photosynthesis) and ultimately entered the food chain via animals and humans.^37^ After plants or animals die, the ^14^C decays over time at a constant rate, which is used to determine the age of the fossil by comparing the measured levels in the sample to the atmospheric levels of ^14^C (Figure 2). This approach was adapted by the Friesen laboratory to determine the age of cardiomyocytes in the human heart.^36,38^ In the 1960s, above-ground nuclear testing released a ^14^C pulse into the atmosphere, although the pulse was limited because all above-ground nuclear tests were halted following the Nuclear Test Ban Treaty (1963).^39^ Therefore, the pulse of ^14^C (by the nuclear tests) was used to demonstrate that approximately 0.5% to 1% new cardiomyocytes were formed each year resulting in the replacement of about half the heart over a lifetime in the average human (Figure 2).^36,38^ These studies verified that the human heart had cardiomyocyte turnover (proliferation) but at a very low rate. Importantly, these results have been further verified by other investigators using animal (mammalian) models.^40^

## Factors and Pathways that Promote Cardiomyocyte Proliferation in the Adult Mammalian Heart

**Figure 3:**
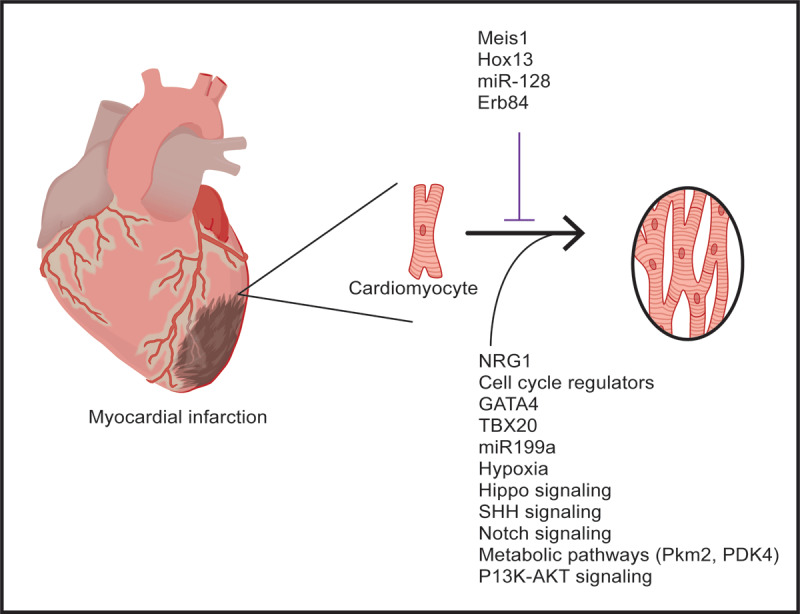
Factors and pathways promote cardiomyocyte proliferation in the adult mammalian heart. Schematics outlining several of the factors, environmental conditions, and pathways that have been reported to promote cardiomyocyte proliferation following injury of the adult mammalian heart. Figure is adapted from references 36-38.

As previously outlined, the invertebrate heart and the neonatal mouse heart have tremendous regenerative capacity due to cardiomyocyte proliferation. These studies as well as the definition of pathways that govern cardiomyocyte proliferation during embryogenesis, have provided an important platform to examine their impact on the cell cycle reentry of adult mammalian cardiomyocytes (Figure 3). Moreover, the carbon dating studies supported the notion that cardiomyocyte turnover occurred but at a limited rate, and intense efforts have attempted to decipher the factors and pathways that promoted cardiomyocyte cell cycle reentry and proliferation as a platform to promote cardiac regeneration. Overexpression of cell cycle activators in the heart has been extensively pursued and shown to promote cardiomyocyte proliferation. Screening strategies using proliferating fetal cardiomyocytes identified CDK4-cyclin D1, CDK1/CDK4/cyclin B1/cyclin D1 as cell cycle regulators that, when overexpressed in rodent or human cardiomyocytes, promoted proliferation.^41^ An independent initiative by the Zhang lab at the University of Alabama at Birmingham and Zangi lab at the Icahn School of Medicine at Mount Sinai demonstrated that cardiomyocyte specific overexpression of the cell cycle regulator, CCND2, in the injured mouse and porcine hearts resulted in increased cardiomyocyte proliferation, decreased infarct size, and improved cardiac function (Figure 3).^42^ This latter study was important as it demonstrated the ability to promote cardiomyocyte proliferation using a transient, nonintegrating delivery system (ie, modRNAs).^42^

Genetic mouse models using gene disruption strategies to delete candidate factors such as NRG1, E2F, and others decreased cardiomyocyte proliferation, whereas transgenic overexpression of factors including: GATA4,^43^ TBX20,^44^ miR-302,^45^ miR199a,^46^ miR-294,^47^ or knockout of miR-128,^48^ Meis1,^27,49,50^ Hox13,^51^ or ErbB4^52^ increased CM proliferation. Studies undertaken in the Institute for Pediatric Research Kühn laboratory at the University of Pittsburg Medical Center defined the growth factor, Neuregulin1 (NRG1),^52^ as one of the first factors that promoted cardiomyocyte proliferation in the mouse and human heart.^52^ Database mining of single cell multi-omics and artificial intelligence have identified new factors and hold tremendous promise for the definition of factors that govern cardiomyocyte proliferation.

Similarly, the modulation of signaling pathways has been shown to induce CM proliferation. For example, the phosphoinositide-3-kinase/protein kinase B (PI3K-AKT) signaling pathway has been shown to control cell cycle activity. Small molecules that activate PI3K-AKT signaling have successfully induced neonatal and adult CM proliferation.^53^ Studies have also shown that the PI3K-AKT pathway may also play a cardioprotective role and/or promote cellular survival, which may be beneficial following an injury.^54^

The Hippo signaling pathway has been shown to have a critical role in regulating the size of an organ during embryogenesis. This pathway relies on the regulation of the phosphorylation status of yes-associated protein, which is a transcriptional coactivator that regulates a number of downstream effectors including cell cycle regulators,^55^ insulin-like growth factor-1,^56^ Wnt^57^ signaling, and others. Studies from several laboratories have demonstrated that inhibition of the pathway promotes cardiomyocyte proliferation. For example, the Martin Heart Lab at the Texas Heart Institute performed LAD ligation-induced cardiac injury in adult mice and pigs, and 2 weeks following the injury they delivered an AAV-9 vector, which knocked down the Hippo pathway gene (Salvador) and demonstrated improved cardiac function (~14% improvement in the ejection fraction), reduced scar size, and cardiomyocyte proliferation.^58,59^ Collectively, these studies emphasized the impact of targeting the border zone surrounding the initial injury, the importance of the Hippo pathway in regulating cell proliferation, and the use of an AAV9 vector to modulate lineage specific pathways (Figure 3).

The Sonic hedgehog pathway (SHH) has been shown to have critical roles during embryogenesis and cancer. The downstream effectors of this pathway are Gli1, Gli2, and Gli3.^18,60^ These transcriptional regulators have been shown to promote cardiomyocyte proliferation and cardiac regeneration following LAD ligation-induced injury in the newt, mouse, and human model systems.^18^ The SHH pathway has also been shown to regulate other pathways including the Wnt/β-catenin,^18,61^ the Notch Pathway,^62^ and others. These latter pathways may also (independent of the SHH pathway) promote cardiomyocyte proliferation. For example, Wnt/β-catenin inhibition has been shown to increase cyclin D, which is associated with cell cycle progression for the cardiomyocytes. Using both genetic mouse models and a modified RNA delivery strategy to promote the SHH pathway, the Garry lab demonstrated improved cardiac function, decreased scar size, and increased cardiomyocyte proliferation following myocardial infarction in the adult mammalian heart (Figure 3).^18^

Oxygenation and the metabolic milieu have been shown to be powerful regulators of cardiomyocyte proliferation. Studies performed by the Sadek laboratory have shown that systemic hypoxemia following LAD ligation to induce cardiac injury robustly activates cardiomyocyte proliferation, decreases fibrosis (scar formation), and improves cardiac function (Figure 3).^49^ Mechanistically, these responses are a result of an induction of Hif1 (and its downstream effectors), decreased oxidative damage, and decreased mitochondrial metabolism. Furthermore, considerable interest has been directed towards an enhanced understanding of the metabolic status of the adult cardiomyocyte and its capacity to reenter the cell cycle and proliferate. For example, the transition of the adult cardiomyocyte from fatty acid utilization to glucose utilization (by deleting pyruvate dehydrogenase kinase 4 or PDK4) in mice has been shown to increase cardiomyocyte proliferation.^63^ Similarly, studies have shown that overexpression of pyruvate kinase muscle isoenzyme 2 (Pkm2) using a lineage-specific modified RNA delivery strategy resulted in increased cardiomyocyte cell division and improved cardiac function.^64^ These studies emphasize the important role of metabolic pathways as regulators of the cardiomyocyte cell cycle reentry and cardiac regeneration.

The above referenced stimulators of cardiomyocyte proliferation are examples of the exciting new strategies that are aimed at promoting cardiomyocyte proliferation during a transient period following an acute injury. Single cell analyses in combination with nonintegrating delivery systems hold tremendous promise for emerging regenerative therapies for cardiovascular diseases.

## Conclusions

Comprehensive analyses of the regenerating heart in lower organisms and the injured neonatal mammalian heart have uncovered important factors and signaling pathways that promote cardiomyocyte proliferation and myocardial repair. Hypoxia, metabolic pathways, cell cycle regulators, transcriptional factors, and signaling pathways have been shown to induce cardiomyocyte proliferation in the adult mammalian heart following injury. The development of viral vectors and modified RNAs holds great promise focused on the targeting of specific lineages, the promotion of cardiomyocyte proliferation, and repair/regeneration of the injured adult heart.

## Key Points

- Lower organisms such as the newt and zebrafish have the capacity for complete repair and regeneration of the injured adult heart.
- The injured neonatal mouse and pig hearts are capable of complete repair due to proliferating cardiomyocytes.
- The adult mammalian heart has approximately 0.5% to 1.0% cardiomyocyte turnover each year.
- Modulation of factors and signaling pathways have been shown to increase cardiomyocyte proliferation and improve cardiac function following injury of the adult mammalian heart.

## CME Credit Opportunity

Houston Methodist is accredited by the Accreditation Council for Continuing Medical Education (ACCME) to provide continuing medical education for physicians.

Houston Methodist designates this Journal-based CME activity for a maximum of *1 AMA PRA Category 1 Credit*™. Physicians should claim only the credit commensurate with the extent of their participation in the activity.

Click to earn CME credit: learn.houstonmethodist.org/MDCVJ-19.5.

## References

[1] Tsao CW, Aday AW, Almarzooq ZI, et al. Heart Disease and Stroke Statistics—2022 Update: A Report From the American Heart Association. Circulation. 2022 Feb 22;145(8):e153-e639. doi: 10.1161/CIR.0000000000001052 35078371

[2] Sanchez PA, O’Donnell CT, Francisco N, et al. Right Ventricular Dysfunction Patterns among Patients with COVID-19 in the Intensive Care Unit: A Retrospective Cohort Analysis. Ann Am Thorac Soc. 2023 Oct;20(10):1465-74. Epub 2023/07/21. doi: 10.1513/AnnalsATS.202303-235OC 37478340 PMC10559129

[3] Lechner A, Herzig JJ, Kientsch JG, et al. Cardiomyopathy as cause of death in Duchenne muscular dystrophy: a longitudinal observational study. ERJ Open Res. 2023 Sep 18;9(5). doi: 10.1183/23120541.00176-2023 PMC10505954 37727676

[4] Kamdar F, Das S, Gong W, et al. Stem Cell-Derived Cardiomyocytes and Beta-Adrenergic Receptor Blockade in Duchenne Muscular Dystrophy Cardiomyopathy. J Am Coll Cardiol. 2020 Mar 17;75(10):1159-74. doi: 10.1016/j.jacc.2019.12.066 32164890 PMC9235061

[5] Wu X, Yang F, Sun L. Computed tomography myocardial perfusion imaging of patients with left ventricular hypertrophy in hypertension: A retrospective study. Clin Exp Hypertens. 2023 Dec 31;45(1):2159426. doi: 10.1080/10641963.2022.2159426 36594488

[6] Chen HHL, Bhat A, Gan GCH, et al. The impact of body mass index on cardiac structure and function in a cohort of obese patients without traditional cardiovascular risk factors. Int J Cardiol Cardiovasc Risk Prev. 2023 Sep 9;19:200211. doi: 10.1016/j.ijcrp.2023.200211 37719420 PMC10502350

[7] Montalvo-Ocotoxtle IG, Rojas-Velasco G, Rodríguez-Morales O, et al. Chagas Heart Disease: Beyond a Single Complication, from Asymptomatic Disease to Heart Failure. J Clin Med. 2022 Dec 7;11(24). doi: 10.3390/jcm11247262 PMC9784121 36555880

[8] Wilsbacher L, McNally EM. Genetics of Cardiac Developmental Disorders: Cardiomyocyte Proliferation and Growth and Relevance to Heart Failure. Annu Rev Pathol. 2016 May 23;11(1):395-419. doi: 10.1146/annurev-pathol-012615-044336 26925501 PMC8978617

[9] Grubb AF, Greene SJ, Fudim M, Dewald T, Mentz RJ. Drugs of Abuse and Heart Failure. J Card Fail. 2021 Nov;27(11):1260-75. doi: 10.1016/j.cardfail.2021.05.023 34133967

[10] Garry DJ, Weiner JI, Greising SM, Garry MG, Sachs DH. Mechanisms and strategies to promote cardiac xenotransplantation. J MilCell Cardiol. 2022 Nov;172:109-19. doi: 10.1016/j.yjmcc.2022.07.013 36030840

[11] Matsukawa R, Okahara A, Tokutome M, et al. A scoring evaluation for the practical introduction of guideline-directed medical therapy in heart failure patients. ESC Heart Fail. 2023 Sep 6. doi: 10.1002/ehf2.14524 PMC10682854 37671603

[12] Garry DJ, Goetsch SC, McGrath AJ, Mammen PP. Alternative therapies for orthotopic heart transplantation. Am J Med Sci. 2005 Aug;330(2):88-101. doi: 10.1097/00000441-200508000-00006 16103789

[13] Garry DJ, Weiner JI, Greising SM, Sachs DH, Garry MG. Xenotransplantation and exotransplantation: Strategies to expand the number of donor organs. Xenotransplantation. 2023 Jan;30(1):e12786. DOI: 10.1111/xen.12786 36367201 PMC13022990

[14] Wang T, Chen X, Wang K, et al. Cardiac regeneration: Pre-existing cardiomyocyte as the hub of novel signaling pathway. Genes Dis. 2024 Mar 24;11(2):747-59. doi: 10.1016/j.gendis.2023.01.031 37692487 PMC10491875

[15] Muralidhar SA, Mahmoud AI, Canseco D, Xiao F, Sadek HA. Harnessing the power of dividing cardiomyocytes. Glob Cardiol Sci Pract. 2013 Nov 1;2013(3):212-21. doi: 10.5339/gcsp.2013.29 24689023 PMC3963758

[16] Salama ABM, Gebreil A, Mohamed TMA, Abouleisa RRE. Induced Cardiomyocyte Proliferation: A Promising Approach to Cure Heart Failure. Int J Mol Sci. 2021 Jul 19;22(14):7720. doi: 10.3390/ijms22147720 PMC8303201 34299340

[17] Singh BN, Doyle MJ, Weaver CV, Koyano-Nakagawa N, Garry DJ. Hedgehog and Wnt coordinate signaling in myogenic progenitors and regulate limb regeneration. Developmental biology. 2012 Nov 1;371(1):23-34. doi: 10.1016/j.ydbio.2012.07.033 22902898 PMC3987681

[18] Singh BN, Koyano-Nakagawa N, Gong W, et al. A conserved HH-Gli1-Mycn network regulates heart regeneration from newt to human. Nat Commun. 2018 Oct 12;9(1):4237. doi: 10.1038/s41467-018-06617-z 30315164 PMC6185975

[19] Singh BN, Koyano-Nakagawa N, Garry JP, Weaver CV. Heart of newt: a recipe for regeneration. J Cardiovasc Transl Res. 2010 Aug;3(4):397-409. doi: 10.1007/s12265-010-9191-9 20559775

[20] Borchardt T, Looso M, Bruckskotten M, Weis P, Kruse J, Braun T. Analysis of newly established EST databases reveals similarities between heart regeneration in newt and fish. BMC Genomics. 2010 Jan 4;11:4. doi: 10.1186/1471-2164-11-4 20047682 PMC2823690

[21] Pohjoismäki JL, Boettger T, Liu Z, Goffart S, Szibor M, Braun T. Oxidative stress during mitochondrial biogenesis compromises mtDNA integrity in growing hearts and induces a global DNA repair response. Nucleic Acids Res. 2012 Aug;40(14):6595-607. doi: 10.1093/nar/gks301 22508755 PMC3413112

[22] Piatkowski T, Braun T. Long-Term Organ Cultures of Newt Hearts. In: Kumar A, Simon A, editors. Salamanders in Regeneration Research: Methods and Protocols. New York, NY: Springer New York; 2015. p. 241-51. 10.1007/978-1-4939-2495-0_19 25740491

[23] de Wit L, Fang J, Neef K, et al. Cellular and Molecular Mechanism of Cardiac Regeneration: A Comparison of Newts, Zebrafish, and Mammals. Biomolecules. 2020 Aug 19;10(9):1204. doi: 10.3390/biom10091204 32825069 PMC7564143

[24] Wang J, Panáková D, Kikuchi K, et al. The regenerative capacity of zebrafish reverses cardiac failure caused by genetic cardiomyocyte depletion. Development. 2011 Aug;138(16):3421-30. doi: 10.1242/dev.068601 21752928 PMC3143562

[25] Soonpaa MH, Kim KK, Pajak L, Franklin M, Field LJ. Cardiomyocyte DNA synthesis and binucleation during murine development. Am J Physiol. 1996 Nov;271(5 Pt 2):H2183-9. doi: 10.1152/ajpheart.1996.271.5.H2183 8945939

[26] Porrello ER, Mahmoud AI, Simpson E, et al. Transient regenerative potential of the neonatal mouse heart. Science. 2011 Feb 25;331(6020):1078-80. doi: 10.1126/science.1200708 21350179 PMC3099478

[27] Mahmoud AI, Kocabas F, Muralidhar SA, et al. Meis1 regulates postnatal cardiomyocyte cell cycle arrest. Nature. 2013 May 9;497(7448):249-53. doi: 10.1038/nature12054 23594737 PMC4159712

[28] Zhu W, Zhang E, Zhao M, et al. Regenerative Potential of Neonatal Porcine Hearts. Circulation. 2018 Dec 11;138(24):2809-16. doi: 10.1161/CIRCULATIONAHA.118.034886 30030418 PMC6301098

[29] Zhao M, Zhang E, Wei Y, Zhou Y, Walcott GP, Zhang J. Apical Resection Prolongs the Cell Cycle Activity and Promotes Myocardial Regeneration After Left Ventricular Injury in Neonatal Pig. Circulation. 2020 Sep;142(9):913-6. doi: 10.1161/CIRCULATIONAHA.119.044619 32866067 PMC8549525

[30] Nakada Y, Zhou Y, Gong W, et al. Single Nucleus Transcriptomics: Apical Resection in Newborn Pigs Extends the Time Window of Cardiomyocyte Proliferation and Myocardial Regeneration. Circulation. 2022 Jun 7;145(23):1744-7. doi: 10.1161/circulationaha.121.056995 35666813 PMC9202233

[31] Garry GA, Antony ML, Garry DJ. Cardiotoxin Induced Injury and Skeletal Muscle Regeneration. Methods Mil Biol. 2016;1460:61-71. doi: 10.1007/978-1-4939-3810-0_6 27492166

[32] Meeson AP, Hawke TJ, Graham S, et al. Cellular and molecular regulation of skeletal muscle side population cells. Stem Cells. 2004;22(7):1305-20. doi: 10.1634/stemcells.2004-0077 15579648

[33] Garry DJ, Meeson A, Elterman J, et al. Myogenic stem cell function is impaired in mice lacking the forkhead/winged helix protein MNF. Proc Natl Acad Sci. 2000 May 9;97(10):5416-21. doi: 10.1073/pnas.100501197 10792059 PMC25843

[34] Hawke TJ, Meeson AP, Jiang N, et al. p21 is essential for normal myogenic progenitor cell function in regenerating skeletal muscle. Am J Phisiol Cell Physiol. 2003 Nov;285(5):C1019-27. doi: 10.1152/ajpcell.00055.2003 12826599

[35] Liu N, Garry GA, Li S, et al. A Twist2-dependent progenitor cell contributes to adult skeletal muscle. Nature Cell Biol. 2017 Mar;19(3):202-13. doi: 10.1038/ncb3477 28218909 PMC5332283

[36] Bergmann O, Bhardwaj RD, Bernard S, et al. Evidence for cardiomyocyte renewal in humans. Science. 2009 Apr 3;324(5923):98-102. doi: 10.1126/science.1164680 19342590 PMC2991140

[37] Graham E, Bergmann O. Dating the Heart: Exploring Cardiomyocyte Renewal in Humans. Physiology (Bethesda). 2017 Jan;32(1):33-41. doi: 10.1152/physiol.00015.2016 27927803

[38] Bergmann O, Zdunek S, Felker A, et al. Dynamics of Cell Generation and Turnover in the Human Heart. Cell. 2015 Jun 18;161(7):1566-75. doi: 10.1016/j.cell.2015.05.026 26073943

[39] Thaul S, Page WF, Crawford H, O’Maonaigh H. Institute of Medicine Committee to Study the Mortality of Military Personnel Present at Atmospheric Tests of Nuclear W. The Five Series Study: Mortality of Military Participants in US Nuclear Weapons Tests. Washington (DC): National Academies Press (US); 2000. 25077238

[40] Lázár E, Sadek HA, Bergmann O. Cardiomyocyte renewal in the human heart: insights from the fall-out. Eur Heart J. 2017 Aug 7;38(30):2333-42. doi: 10.1093/eurheartj/ehx343 28810672 PMC5837331

[41] Mohamed TMA, Ang YS, Radzinsky E, et al. Regulation of Cell Cycle to Stimulate Adult Cardiomyocyte Proliferation and Cardiac Regeneration. Cell. 2018 Mar 22;173(1):104-16.e12. doi: 10.1016/j.cell.2018.02.014 29502971 PMC5973786

[42] Sun J, Wang L, Matthews RC, et al. CCND2 Modified mRNA Activates Cell Cycle of Cardiomyocytes in Hearts With Myocardial Infarction in Mice and Pigs. Circ Res. 2023 Sep;133(6):484-504. doi: 10.1161/circresaha.123.322929 37565345 PMC10529295

[43] Malek Mohammadi M, Kattih B, Grund A, et al. The transcription factor GATA4 promotes myocardial regeneration in neonatal mice. EMBO Mol Med. 2017 Feb;9(2):265-79. doi: 10.15252/emmm.201606602 28053183 PMC5286367

[44] Lu F, Langenbacher A, Chen JN. Tbx20 drives cardiac progenitor formation and cardiomyocyte proliferation in zebrafish. Dev Biol. 2017 Jan 15;421(2):139-48. doi: 10.1016/j.ydbio.2016.12.009 27940156 PMC5226859

[45] Barroso-delJesus A, Romero-López C, Lucena-Aguilar G, et al. Embryonic stem cell-specific miR302-367 cluster: human gene structure and functional characterization of its core promoter. Mol Cell Biol. 2008 Nov;28(21):6609-19. doi: 10.1128/mcb.00398-08 18725401 PMC2573233

[46] Gabisonia K, Prosdocimo G, Aquaro GD, et al. MicroRNA therapy stimulates uncontrolled cardiac repair after myocardial infarction in pigs. Nature. 2019 May;569(7756):418-22. doi: 10.1038/s41586-019-1191-6 31068698 PMC6768803

[47] Borden A, Kurian J, Nickoloff E, et al. Transient Introduction of miR-294 in the Heart Promotes Cardiomyocyte Cell Cycle Reentry After Injury. Circulation Res. 2019 Jun 21;125(1):14-25. doi: 10.1161/CIRCRESAHA.118.314223 30964391 PMC6586499

[48] Huang W, Feng Y, Liang J, et al. Loss of microRNA-128 promotes cardiomyocyte proliferation and heart regeneration. Nat Commun. 2018 Feb 16;9(1):700. doi: 10.1038/s41467-018-03019-z 29453456 PMC5816015

[49] Simsek T, Kocabas F, Zheng J, et al. The distinct metabolic profile of hematopoietic stem cells reflects their location in a hypoxic niche. Cell Stem Cell. 2010 Sep 3;7(3):380-90. doi: 10.1016/j.stem.2010.07.011 20804973 PMC4159713

[50] Mahmoud AI, Canseco D, Xiao F, Sadek HA. Cardiomyocyte cell cycle: Meis-ing something? Cell Cycle. 2014;13(7):1057-8. doi: 10.4161/cc.28379 24603411 PMC4013154

[51] Nguyen NUN, Canseco DC, Xiao F, et al. A calcineurin-Hoxb13 axis regulates growth mode of mammalian cardiomyocytes. Nature. 2020 Jun;582(7811):271-6. doi: 10.1038/s41586-020-2228-6 32499640 PMC7670845

[52] Bersell K, Arab S, Haring B, Kuhn B. Neuregulin1/ErbB4 signaling induces cardiomyocyte proliferation and repair of heart injury. Cell. 2009 Jul 23;138(2):257-70. doi: 10.1016/j.cell.2009.04.060 19632177

[53] Beigi F, Schmeckpeper J, Pow-Anpongkul P, et al. C3orf58, a novel paracrine protein, stimulates cardiomyocyte cell-cycle progression through the PI3K-AKT-CDK7 pathway. Circulation Res. 2013 Aug 2;113(4):372-80. doi: 10.1161/circresaha.113.301075 23784961 PMC3870268

[54] Song H-P, Chu Z-G, Zhang D-X, Dang Y-M, Zhang Q. PI3K–AKT Pathway Protects Cardiomyocytes Against Hypoxia-Induced Apoptosis by MitoKATP-Mediated Mitochondrial Translocation of pAKT. Cell Physiol Biochem. 2018;49(2):717-27. doi: 10.1159/000493037 30165359

[55] Gründl M, Walz S, Hauf L, et al. Interaction of YAP with the Myb-MuvB (MMB) complex defines a transcriptional program to promote the proliferation of cardiomyocytes. PLoS Genet. 2020 May 29;16(5):e1008818. doi: 10.1371/journal.pgen.1008818 32469866 PMC7286521

[56] Xin M, Kim Y, Sutherland LB, et al. Regulation of insulin-like growth factor signaling by Yap governs cardiomyocyte proliferation and embryonic heart size. Sci Signal. 2011 Oct 25;4(196):ra70. doi: 10.1126/scisignal.2002278 22028467 PMC3440872

[57] Bergmann MW, Kühl M. WNT Signaling in Adult Cardiac Hypertrophy and Remodeling. Circulation Res. 2010 Nov 12;107(10):1198-208. doi: 10.1161/CIRCRESAHA.110.223768 21071717

[58] Liu S, Li K, Wagner Florencio L, et al. Gene therapy knockdown of Hippo signaling induces cardiomyocyte renewal in pigs after myocardial infarction. Sci Transl Med. 2021 Jun 20;13(600):eabd6892. doi: 10.1126/scitranslmed.abd6892 34193613 PMC9476348

[59] Leach JP, Heallen T, Zhang M, et al. Hippo pathway deficiency reverses systolic heart failure after infarction. Nature. 2017 Oct 12;550(7675):260-4. doi: 10.1038/nature24045 28976966 PMC5729743

[60] Dunaeva M, Waltenberger J. Hh signaling in regeneration of the ischemic heart. Cell Mol Life Sci. 2017 Oct;74(19):3481-90. doi: 10.1007/s00018-017-2534-9 28523343 PMC5589787

[61] Willems E, Spiering S, Davidovics H, et al. Small-molecule inhibitors of the Wnt pathway potently promote cardiomyocytes from human embryonic stem cell-derived mesoderm. Circulation Res. 2011 Aug 5;109(4):360-4. doi: 10.1161/circresaha.111.249540 21737789 PMC3327303

[62] Gude NA, Emmanuel G, Wu W, et al. Activation of Notch-Mediated Protective Signaling in the Myocardium. Circulation Res. 2008 May 9;102(9):1025-35. doi: 10.1161/CIRCRESAHA.107.164749 18369158 PMC3760732

[63] Cardoso AC, Lam NT, Savla JJ, et al. Mitochondrial Substrate Utilization Regulates Cardiomyocyte Cell Cycle Progression. Nat Metab. 2020 Feb;2(2):167-78. PMID: 32617517 32617517 PMC7331943

[64] Magadum A, Singh N, Kurian AA, et al. Pkm2 Regulates Cardiomyocyte Cell Cycle and Promotes Cardiac Regeneration. Circulation. 2020 Apr 14;141(15):1249-65. doi: 10.1161/circulationaha.119.04067 32078387 PMC7241614

